# Effect of Hatch Spacing on Melt Pool and As-built Quality During Selective Laser Melting of Stainless Steel: Modeling and Experimental Approaches

**DOI:** 10.3390/ma12010050

**Published:** 2018-12-24

**Authors:** Zhichao Dong, Yabo Liu, Weibin Wen, Jingran Ge, Jun Liang

**Affiliations:** 1 Institute of Advanced Structure Technology, Beijing Institute of Technology, Beijing 100081, China; zc339580@126.com (Z.D.); yabo0019@163.com (Y.L.); 2 Beijing Key Laboratory of Lightweight Multifunctional Composite Materials and Structures, Beijing Institute of Technology, Beijing 100081, China; 3 School of Civil Engineering, Central South University, Changsha, 410083, China; wenwbin@126.com; 4 State Key Laboratory of Explosion Science and Technology, Beijing Institute of Technology, Beijing 100081, China

**Keywords:** hatch spacing, overlap rate, selective laser melting, stainless steel microstructure, relative density

## Abstract

In this study, a combined simulation and experimental approach is utilized to investigate the influence of hatch spacing on the microstructure and as-built quality of 316L stainless steel (SS) samples fabricated by selective laser melting (SLM). A three-dimensional finite element model (FEM) is employed to investigate heat transfer and melt pool during the SLM of 316L SS. The phase transformation and variation of the thermo-physical properties of the materials are considered in this model. The effects of hatch spacing (H) on the temperature field, microstructure and melt pool size, overlap rate, surface quality, and relative density during the SLM of 316L SS are investigated. The simulated results indicate that, as the hatch spacing increases, the depth increases and the width of the melt pool decreases. Meanwhile, with the increase of hatch spacing, the simulated temperature of the subsequent tracks falls below the melting temperature of the first track. Moreover, the microstructures were found to coarsen with the increasing hatch spacing due to the reduced cooling rate. The optimized hatch spacing and overlap rate between adjacent tracks were obtained from numerical simulations. Simulation results illustrate that, when the optimized hatch spacing of 100 μm is adopted, fully dense parts with a smooth surface can be fabricated by SLM, thus experimentally validating the simulation results.

## 1. Introduction

Selective laser melting (SLM) is a rapidly thriving additive manufacturing (AM) process to fabricate complex geometrical metal parts [1,2]. A study has shown that as metal powders reach their melting and solidification temperatures by heating or cooling during SLM, phase change (i.e., melting) occurs [3,4]. However, the physical and chemical metallurgical processes in SLM are complex and involve heat transfer, heat convection, and radiation [5,6,7,8]. Due to the particularity of SLM, it is difficult to observe the thermal behavior, phase transitions, and melt-pool behavior directly because they strongly depend on the laser process parameters, such as the hatch spacing, scanning speed, and laser power etc. [9,10,11,12]. Alsalla et al. [13] investigated the density, surface quality, microstructure, and mechanical properties of the components of the SLM parts made at different building directions. Kurzynowski et al. [14] reported the influences of laser power input and scanning and building strategies on the microstructure, texture, and tensile properties of SLM-produced 316L SS through experiments. In fact, different hatch spacings resulted in different heat-transfer behaviors, surface qualities, and overlap rates [15,16,17]. In addition, the ultimate density and building rate were determined by the hatch spacing, as the parts are built in a layer-wise manner, and each layer is manufactured in the track-wise fashion [18,19]. After comparing all the other process parameters, a higher building rate was obtained by increasing the hatch spacing. However, further increasing the hatch spacing causes insufficient melting of particles and high porosity. Therefore, to achieve optimized SLM-processed components, research efforts are devoted to determining the impact of the hatch spacing as well as the melting/solidification mechanism. Owing to the fast-moving laser source, quick melting and solidification of the metal powder, and the exceedingly small melt pool size, it is difficult to determine the abrupt temperature variation during transient heat transfer through experimental measurements. Fortunately, the finite element method provides a unique insight into understanding the influence of the hatch spacing on the temperature distribution, melt pool size, relative density, and overlap rate between neighboring tracks during SLM.

To date, several experimental and finite element simulation studies of the thermal behavior during SLM have been reported. Su et al. [19] have optimized the inter-layer overlapping regime and track space to obtain SLM-processed 316L SS parts with a high relative density. Yadroitsev et al. [20] investigated the influence of hatch spacing on surface quality during SLM of 904L SS. They found that a gap between single vectors appeared at a hatch spacing greater than 120 μm, which means the quantity of powder was insufficient. Wen et al. [21] reported the effect of processing parameters on the densification behavior of SLM-produced pure Zn metal parts, and obtained high density in a reasonable process window. AlMangour et al. [22] studied the densification behavior, microstructural evolution, and compression properties of TiC/316L nanocomposites processed using various processing parameters. Antony et al. [23] focused on the effect of process parameters on the thermal transfer and melt pool size during SLM of 316L SS using an finite element model (FEM) and single track experiments. Yu et al. [24] studied the fluid flow behavior and heat transfer of the melt pool with mesoscopic simulations. They found that high and low laser powers have a significant influence on the quality of the rough surface. Wu et al. [25] developed a random powder-packing mesoscale model with multiple-diameter particles by considering the influence of material evaporation on the behavior of the melt pool. They observed that the process parameter in SLM has an important effect on the thermal behavior, melt pool size, surface quality, and relative density. The melting process usually involves multiple tracks, which interact with each other. However, most powder bed models can only simulate the melting process within a single track. Currently, a very limited amount of the reported research is devoted to studying the effects of large hatch spacing on the melt pool size and thermal transfer in SLM. Moreover, experiment methods for process parameters’ optimization not only have high time costs, but also have some difficulties in determining the temperature distribution during SLM. The development of a feasible method for the selection of process parameters for SLM is thus urgently required.

In this work, a combined simulation and experimental approach is developed to investigate the influence of hatch spacing on microstructure and as-built quality of 316L SS samples fabricated by SLM. A three-dimensional FEM for macroscopic simulations is employed to assess the influence of large hatch spacings on the SLM of 316L SS using the commercial software (ABAQUS) (SIMULIA, Providence, RI, USA). The latent heat and the variation of the thermo-physical properties of the 316L SS powder are considered. In addition, the temperature profiles, microstructure, melt pool size, overlap rate, and sintering at the bottom of the scanning tracks are simulated. With simulation analysis, an optimized value of the hatch spacing is determined, which yields a relatively flat sintering condition and an appropriate overlap rate between adjacent tracks. The optimized process parameters obtained from the simulations are used in an experimental study, and parts with a dense and smooth surface were fabricated by SLM, which experimentally verifies the simulated findings. These results show the effectiveness of using experimentally verified simulations to investigate the useful information about process parameters while significantly reducing the need for experimental analysis.

## 2. Materials and Methods

### 2.1. Simulation

**Figure 1:**
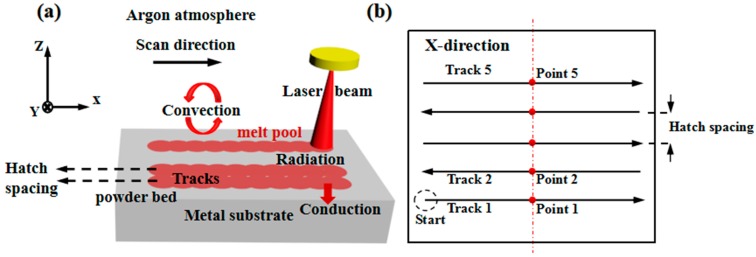
Schematic of the SLM process: (**a**) Physical model of SLM and (**b**) the scanning strategy.

In this model, the following assumptions are made to further simplify the SLM process: (1) The surface source is used on the powder bed surface. (2) The powder material is assumed to be a continuous medium. (3) The vaporization and solid-gas coupling are ignored. Figure 1a depicts a schematic of the SLM process. The environment consists of an argon atmosphere, and a 50 μm layer of 316L SS powder is deposited on the metal substrate. Part of the heat can be dissipated by convection and radiation when the surface of the powder bed is irradiated with a laser beam. The remaining energy of the laser is absorbed by the powder. As a result, localized melting and rapid heating occur, and metallurgical bonds between adjacent tracks are formed when the melt pool has migrated far from the Gaussian surface heat source. Heat conduction within the metal substrate and powder particles, laser radiation to the powder bed, and heat convection between the boundaries of the powder layer and the chamber atmosphere are the three main heat transfer mechanisms of SLM. Figure 1b shows the scanning protocol and the five scanning tracks. The three-dimensional numerical model, with dimensions of 1 mm × 1.25 mm × 0.3 mm, and the powder substrate is discretized with 12.5 μm elements of 8-node linear heat transfer brick. The midpoints of scanning tracks 1, 2, and 5 represent points 1, 2, and 5, respectively. The used material properties and processing parameters are listed in Table 1. The thermo-physical parameters of 316L SS (EOS plc, Munich, Germany) are listed in Table 2.

The governing equations of the three-dimensional heat transfer process are expressed as follows:

(1) $$\rho c\frac{\partial T}{\partial t}=Q+\frac{\partial }{\partial x}\left(k\frac{\partial T}{\partial x}\right)+\frac{\partial }{\partial y}\left(k\frac{\partial T}{\partial y}\right)+\frac{\partial }{\partial z}\left(k\frac{\partial T}{\partial z}\right)$$

where *ρ* is the density of 316L SS; *c* is the specific heat; *T* is the temperature; *t* is the interaction time; *Q* (*x*,*y*,*z*,*t*) is the heat generated; and *k* is the effective thermal conductivity of the powder bed. The initial temperature throughout the powder bed is defined by Equation (2).

(2) $$T{\left(x,y,z,t\right)}_{t=0}=293K$$

The boundary conditions at the surface of the powder bed were defined similarly to Hussein’s methods [28]:

(3) $$-k{\left(\frac{\partial T}{\partial z}\right)}_{z=0}=\frac{2AP}{\pi \omega^{2}}\exp\left(-\frac{2r^{2}}{\omega^{2}}\right)-h(T-T_{s})-\sigma \varepsilon \left(T^{4}-{T_{s}}^{4}\right)$$

where *ω* is the beam radius at which the heat flux is e-2 times that of the laser beam center; *A* is the absorption rate of the powder bed; *r* is the distance of a point on the surface from the center of the laser beam; *T_s_* is the ambient temperature; *h* is the heat transfer coefficient of thermal convection; *σ* is the Stefan-Boltzmann constant; and *ε* is the emissivity.

To simulate the temperature field more accurately, the user-written subroutine (UMATHT), was implemented for the variation of thermo-physical parameters of the powder and solid materials. Assuming the powders are spherical, the effective thermal conductivity, *k_eff_*, of the powder layer can be described as [29]:

(4) $$\frac{k_{eff}}{k_{f}}=(1-\sqrt{1-\varphi })\left(1+\frac{\varphi k_{r}}{k_{f}}\right)+\sqrt{1-\varphi }\left[\frac{2}{1-\frac{k_{f}}{k_{s}}}\left(\frac{1}{1-\frac{k_{f}}{k_{s}}}\ln\left(\frac{k_{s}}{k_{f}}\right)-1\right)+\frac{k_{r}}{k_{f}}\right]$$

where *k_f_* and *k_s_* are the thermal conductivity of the gas and solid around the powder and substrate, respectively; *k_r_* and *φ* are the thermal conductivity and porosity of the powder bed, which can be described by Equations (5) and (6), respectively:

(5) $$kr=4B\sigma Tp^{3}Dp$$

where *D_p_* is the average diameter of the powder particles; *T_p_* is the temperature of the particles; *σ* is the Stefan-Boltzmann constant; and *B* is a view factor, which is set as 1/3.

(6) $$\varphi =\frac{\rho_{s}-\rho_{p}}{\rho_{s}}$$

where *ρ_s_* and *ρ_p_* are the density of the solid and metal powder, respectively. The porosity of the tapped powder bed, *φ*, is set as 0.45 [28].

By considering the powder bed as a mixture of solid particles (316L SS) and gas phases, the volumetric heat capacity of the powder bed can be expressed as:

(7) $$\rho_{p}C_{p}=\left(1-\varphi \right)\left(\rho_{s}C_{s}\right)+\varphi \left(\rho_{g}C_{g}\right)$$

where *φ* is the porosity of the powder bed, and *ρ_g_* is the density of the gas phase. In addition, *Cp*, *Cs*, and *Cg* are the specific heat of the powder bed, solid (316L SS), and gas (air), respectively. In general, the specific heat of a gas is much lower than that of a solid. According to Equation (7), the specific heat of the powder bed approaches to the specific heat of a solid.

### 2.2. Experimentation

**Figure 2:**
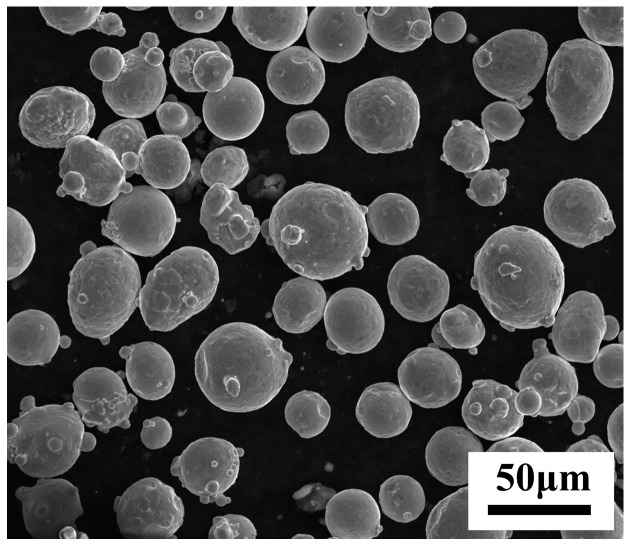
SEM image of the starting powders.

316L SS powder (supplied by EOS plc, Munich, Germany) with an average particle diameter of 45 μm was used as the initial material, and the nominal chemical composition is given in Table 3. The scanning electron micrograph of the 316L SS powder is shown in Figure 2. The samples were fabricated with the EOSINT M280 system (EOS plc, Munich, Germany), which contains an available building space with a volume of 250 mm × 250 mm × 325 mm. The 316L SS samples for the metallographic tests were sliced, ground, polished, and then etched using a reagent (3 mL HCl, 1 mL HNO_3_, and 96 mL H_2_O) for 10 s. The microstructures were observed with a Leica DM-4000M metallographic microscope (Leica, Wetzlar, Germany). The surface morphologies of the samples were observed with an FEG 250 scanning electron microscope (SEM) (FEI, Hillsboro, OR, USA). The surface roughness of SLM-processed samples was measured with a JB-4C precision roughness instrument (SHjingmi plc, Shanghai, China). The relative density of the SLM parts was measured by the Archimedes method. As shown in Table 1, the SLM process parameters in the simulations are the same as those in the experiments.

## 3. Results and Discussion

### 3.1. Temperature Distribution

**Figure 3:**
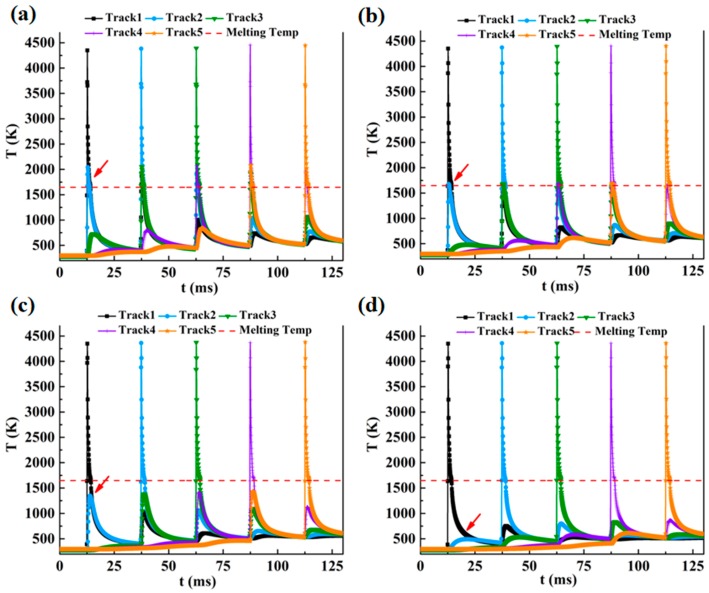
Cyclic melting and cooling of the scanning process of the five tracks for different hatch spacings: (**a**) 75 μm, (**b**) 100 μm, (**c**) 150 μm, and (**d**) 200 μm.

The numerical results of the temperature distribution at the midpoint of each track for different hatch spacings are shown in Figure 3. The peak point of the temperature curve indicates that the start of each laser scan track causes the formation of a layer. As seen in Figure 3a,b, the hatch spacing determines the overlap rate between adjacent tracks, and some sections (red arrow) were exposed to the second scan and melted twice to combine neighboring tracks and form a dense layer. In Figure 3a,b, the temperature in the first track exceeds the melting temperature when the second track was being scanned. As the hatch spacing increased, the temperature of the subsequent tracks fell below the melting temperature, as seen in Figure 3c,d. The peak temperature of the subsequent track was slightly higher than that of the previous scanning track for all hatch spacings (see Figure 3). The peak temperature at the end of the fifth track was calculated to be 4447, 4403, 4379, and 4359 K, for a hatch spacing of 75, 100, 150, and 200 μm, respectively. As shown in Figure 3, the temperature reached a minimum in the first track because of the low thermal conductivity and temperature of the powder bed. After the first track, the powder bed was preheated by the previous scanning track, and, as a result, the peak temperature increased. Increasing the hatch spacing reduced the maximum temperature, overlap rate, and heat accumulation associated with the surface roughness and relative density of the SLM-processed samples.

### 3.2. Melt pool Size

**Figure 4:**
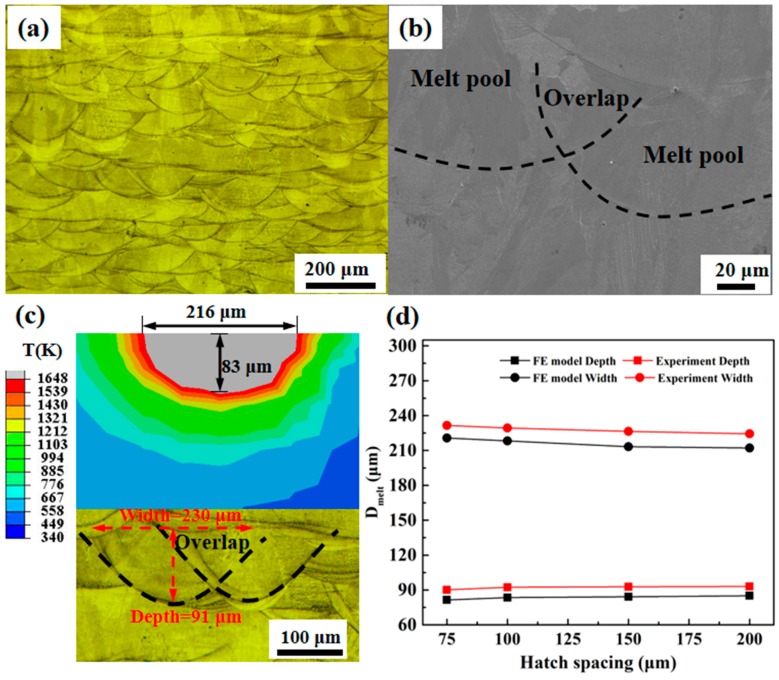
(**a**) Optical and (**b**) SEM representative images of the lateral section of the samples; (**c**) simulated (upper) and experimental (under) melt pool size for a hatch spacing of 100 μm, and (**d**) the results of simulation and experimental: Width/depth of the melt pool for different hatch spacings.

The optical microstructure from the lateral section (parallel to build-direction) is shown in Figure 4a. The as-fabricated samples presented clear solidification tracks on the macro-scale. In Figure 4a,b, the scanning laser beam generated periodic melt pools in each layer, which appear like many aligned welding beads. Their boundaries became well noticeable after etching. The depth and width of the melt pool obtained both numerically and experimentally are shown in Figure 4c,d. The area above the melting temperature (1648 K) of the metal powder bed indicates the melt region, and the size of the melt pool was determined. A picture of the calculated melt pool width and depth is shown in Figure 4c. The melt pool is expressed as the area of the powder bed where the temperature was above the melting point, and is depicted in gray. For a spacing, H, of 100 μm, the width and depth of the gray region were 216 and 83 μm, respectively. Similar data were obtained in previous studies, such as Wang et al. [30], in which the heat transfer and melt pool size during SLM were simulated. To assess the reliability of the FEM model, the melt pool width and depth obtained experimentally were compared with the numerically determined results, which were 230 and 91 μm, respectively, and are therefore consistent with the simulation data.

To validate the results of the proposed model, the width and depth of different hatch spacings (75, 150, and 200 μm) were obtained by simulations and experimentally compared. Figure 4d shows the high similarity between the simulated and experimentally measured width and depth of the melt pool for different hatch spacings, which further confirms the reliability of the model. As the hatch spacing increased from 75 to 200 μm, the simulated width of the melt pool decreased from 221 to 212 μm and the depth increased from 81.4 to 85.2 μm. The experimental results show that when the hatch spacing increased from 75 to 200 μm, the measured width of the melt pool decreased from 231.5 to 224 μm and the depth increased from 90 to 93.5 μm. As seen in Figure 4, the deviation of the FEM model from the experiment was less than 9%. It is worth noting that although that some factors (latent heat of melting and thermal effects) of the SLM process were considered in the model, others (flow behavior) have been simplified. As noted by Ding et al [31], instabilities of the melt pool owing to the velocity field and liquidus duration can change the size of the melt pool.

### 3.3. Microstructure

**Figure 5:**
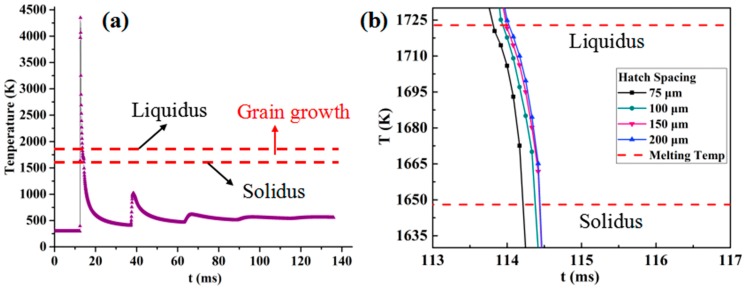
(**a**) FEM temperature-time curve of 316L SS samples simulated based on their optimized parameters and (**b**) its magnified temperature-time curves between the solidus and liquidus for different process parameters.

As mentioned in Section 3.1 and Section 3.2, the cyclic melting and cooling curves and melt pool dimension are discussed. According to the works of Casati et al [32], the fine equiaxed grains are achieved from the melt pool center. In addition, the epitaxial growth of new grains from remelted zones occurred in melt pool boundaries due to the remelted zones. Based on the former results, the temperature-time curve and cooling rate were observable from the simulation as presented in Figure 5. The liquidus and solidus of 316L SS are plotted in Figure 5a. There was a discrepancy in the cooling rates calculated from the gradients of the curves in Figure 5b between the liquidus and solidus. Their value depended on process parameters, including the laser input, scanning speed, and hatch spacing. The relationship between the temperature gradient (G) and the growth rate (R) in prediction of the cellular dendrite size of an SLM-ed austenitic SS specimen was mentioned by Li et al. [33]. The cellular dendrite size can in turn be predicted by the G × R value, which is also the cooling rate (Ṫ). For 316L SS, the cooling rate, Ṫ, and microstructure size, λ, can be predicted mathematically with the relation, λ = 80Ṫ − 0.33, as reviewed by Yadroitsev et al. [34]. Using this empirical equation, we could estimate the grain size based on the cooling rate.

**Figure 6:**
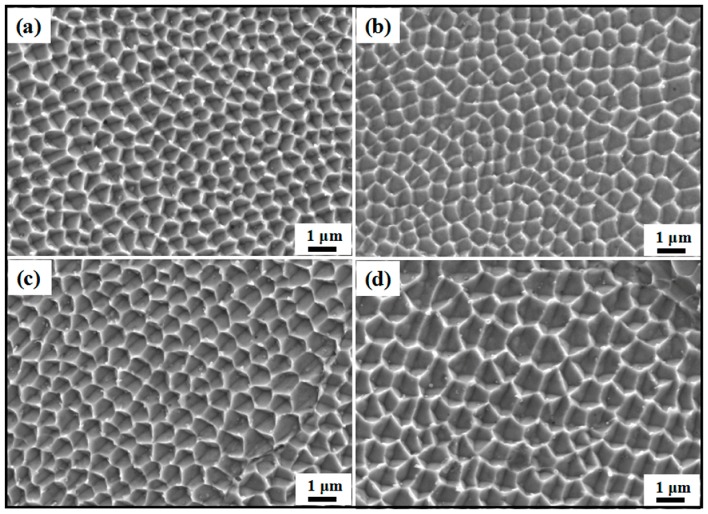
High magnification SEM images of cellular dendrites: (**a**) 75 μm, (**b**) 100 μm, (**c**) 150 μm, and (**d**) 200 μm.

In this work, the different cooling rates were calculated from FEM due to the various hatch spacings. As described in Section 3.1, the maximum temperature was acquired, when the hatch spacing was 75 μm. This implies that when the material can reach a higher maximum temperature in SLM, it is also likely to reach a higher heat gradient and cooling rate. From Table 4, it is shown that the microstructure sizes should increase with the addition of hatch spacing due to the reduced cooling rates. Figure 6 presents the SEM micrographs of built samples at higher magnifications. The average cell sizes of on the SEM image can be approximated with methods used from the works of Ma et al. [35]. As listed in Table 4, the simulation results were in favor of the experimental values.

### 3.4. Overlap Rate

**Figure 7:**
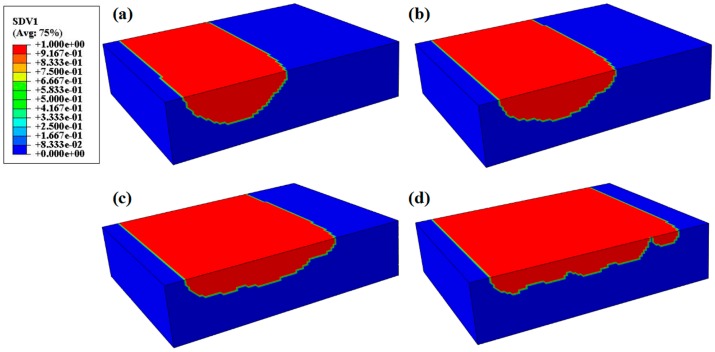
The solidified development at end of the process: (**a**) 75 μm, (**b**) 100 μm, (**c**) 150 μm, and (**d**) 200 μm.

The overlap rate is represented by a percentage; it indicates the areas influenced by repeated melting with the laser beam and is related to the hatch spacing. The hatch spacing determines the overlap rate of the subsequent tracks, and some regions were irradiated by multiple laser scans and melted twice due to the different overlaps [36,37]. Figure 7 shows the solidified development at the end of the fifth track for several cases of 75, 100, 150, and 200 μm hatch spacing. The solution-dependent state variables (SDV1) indicates the melted and solidified development of the metal powders. Therefore, the discrepancy in the melting area can be distinguished at the end of the fifth track. In Figure 7a,b, the depth and overlap rate of the solid zone between the tracks can form a fully dense layer. The insufficient overlap zone and relatively shallower depth of the melt pool were observed due to the increased hatch spacing, as shown in Figure 7c,d.

**Figure 8:**
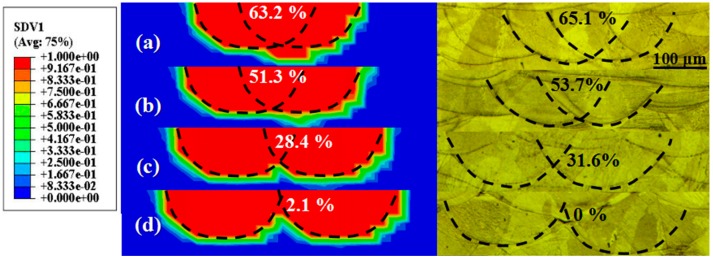
Simulated (left) and experimental (right) overlap rate between adjacent tracks for various hatch spacings: (**a**) H = 75 μm, (**b**) H = 100 μm, (**c**) H = 150 μm, and (**d**) H = 200 μm.

The simulated (left) and experimental (right) results of the overlap rate between adjacent tracks for various hatch spacings are depicted in Figure 8. As seen in Figure 8, the overlap rate decreased with increasing hatch spacing. At the hatch spacing of 75 μm, the maximum overlap rate calculated with the FEM was 63.2%, and the experimental value was approximately 65.1% (Figure 5a). Moreover, the simulation results show the existence of local over-sintering between adjacent tracks. Qiu et al. [18] reported that a relatively high manufacturing temperature was achieved with relatively low hatch spacings, resulting in excessive fusion of the metal powder and the solid. The size of the repeated melting regions between adjacent tracks increased, and ultimately a comparatively higher overlap rate and corresponding sintering conditions were developed. When the hatch spacing increased to 100 μm, the simulated and experimental results in Figure 8b show a good metallurgical combination. At even higher hatch spacings of 150 and 200 μm, regions of insufficient melting exist between neighboring tracks, as seen in Figure 8c,d. The calculated overlap rate decreased to 28.4% and 2.1%, and the experimental rate was approximately 31.6% and 0% (Figure 5c,d, respectively). When large hatch spacings were used for SLM, regions of insufficient melting and pores arose between neighboring tracks, owing to the decrease in heat accumulation. According to the above results, the hatch spacing significantly influences the sintering and overlap rate between adjacent tracks. 

As a result, a reasonable value of the hatch spacing of 100 μm was obtained by the simulation, which yields relatively flat sintering conditions and an appropriate overlap rate (approximately 54%) between adjacent tracks. In the next section, the numerically optimized process parameters were applied experimentally. Furthermore, the other process parameters (hatch spacing of 75, 150, and 200 μm) were also investigated experimentally.

### 3.5. Surface Roughness

**Figure 9:**
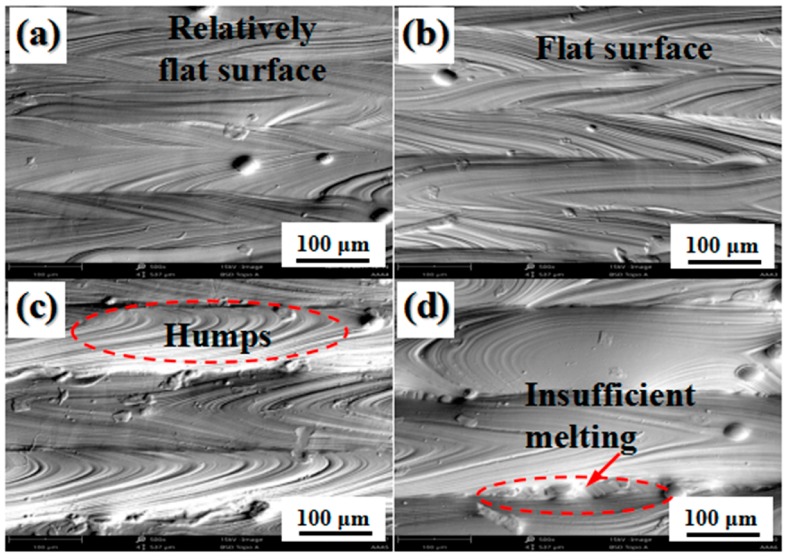
Morphology of the top surface of SLM-processed 316L SS parts for different hatch spacings: (**a**) H = 75 μm, (**b**) H = 100 μm, (**c**) H = 150 μm, and (**d**) H = 200 μm.

**Figure 10:**
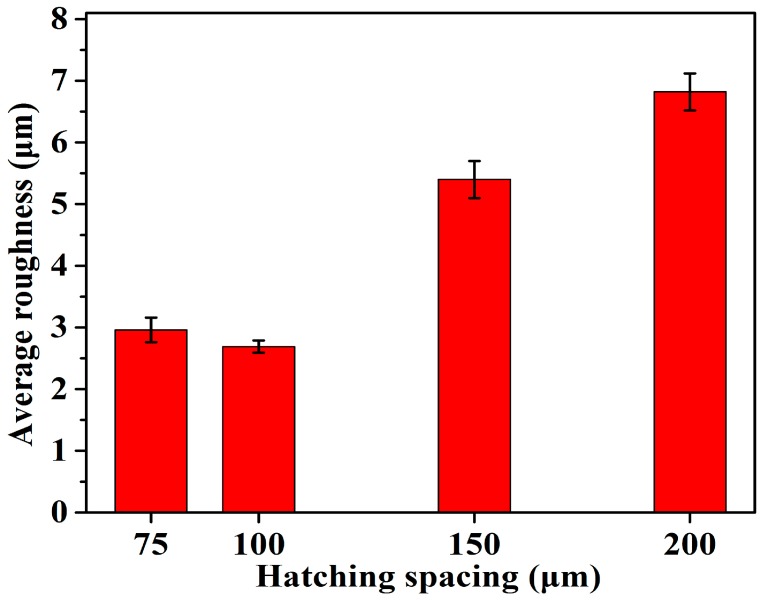
Average roughness of the top surface of the samples for different hatch spacings: (**a**) H = 75 μm, (**b**) H = 100 μm, (**c**) H = 150 μm, and (**d**) H = 200 μm.

Figure 9 shows the top surface morphology of the SLM-processed 316L SS samples for different hatch spacings. A detailed roughness of the top surface of the samples can be seen in Figure 10. The humps and regions of insufficient melting between adjacent tracks changed as the hatch spacing increased from 75 to 200 μm. At the track spacing of 75 μm, a relatively flat surface was observed, as shown in Figure 6a, resulting in a comparatively low surface roughness of 2.96 μm (Figure 10). As the hatch spacing increased to 100 μm, a flat and dense surface without any obvious humps or defects was produced, as seen in Figure 9b, and the sample showed a lower surface roughness of 2.68 μm (Figure 10) compared to the other samples. Because of the sufficient heat accumulation, the melt regions were solidified and subsequently faultlessly filled with blank regions between adjacent tracks. The top surface of the sample showed a number of rippled micro-humps as the hatch spacing increased to 150 μm, as seen in Figure 9c. The average surface roughness reached 5.4 μm (Figure 10). As the hatch spacing further increased to 200 μm, the melted regions were barely filled with blank regions between the scanning tracks, resulting in a relatively large average surface roughness of 6.8 μm (Figure 10), which may be attributed to the low temperature produced by the low heat accumulation and the limiting amount of generated non-melted particles (Figure 9d). As shown in Figure 9 and Figure 10, using the selected process parameter (hatch spacing of 100 μm), a 316L SS sample with a smooth surface with an average surface roughness of 2.68 μm was formed.

These results reveal that the surface quality of SLM-fabricated components mainly depends on the hatch spacing. When the hatch spacing increased, the energy absorption of the powder bed in the blank regions between adjacent scanning tracks decreased, resulting in poor metallurgical bonding in the samples. In addition, Ventola et al. [38] have reported that the surface roughness affects the heat transfer and surface emissivity. A rough surface easily causes regions of insufficient melting, and defects are formed in the newly fabricated layer, compared with relatively low-roughness surfaces. Thus, it is reasonable to conclude that appropriate hatch spacings (such as 100 μm) should be favorable to an SLM process that results in a flat, dense, and coherent metallurgically bonded surface and ultimately improves the mechanical properties of the parts.

### 3.6. Relative Density

**Figure 11:**
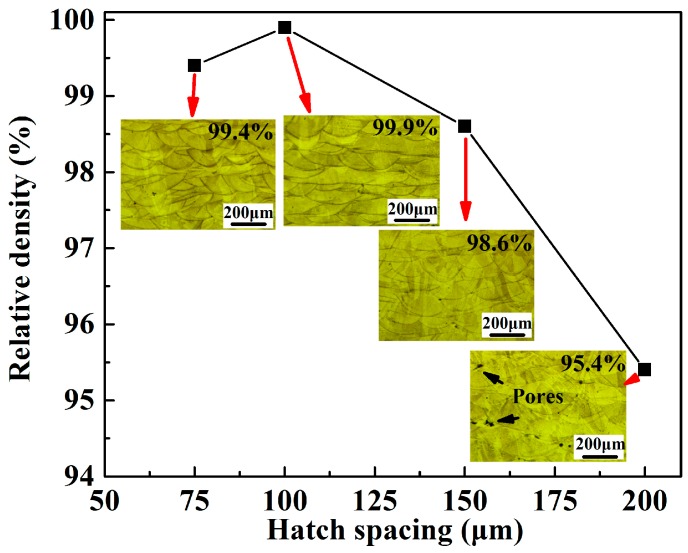
Effect of hatch spacing on the densification rate and cross-sectional microstructure of the SLM-processed 316L SS samples.

Figure 11 displays the effects of the hatch spacing on the relative density of the SLM-fabricated 316L SS specimens. The results indicate that the specimens with a hatch spacing of 75 and 100 μm exhibited a similar relative density of 99.4% and 99.9%, respectively. When the hatch spacing increased to 150 μm, small pores were observed on the samples. Similarly, upon increasing the hatch spacing to 200 μm, the pores of the samples became larger than the pores of other samples. Simultaneously, the number of pores increased (Figure 11). The relative density dropped to 98.6% and 95.4%, when the hatch spacing was 150 and 200 μm, respectively. If H exceeded 100 μm, the width of the new track did not fully cover the hatch spacing distance, resulting in the lack of fusion between tracks. The relatively lower H of 75 μm did not show a positive effect on the further improvement of densification, but reduced the build efficiency instead. AlMangour et al. [39] studied the thermal behavior of the molten pool, microstructural evolution, and tribological performance of SLM-processed TiC/316L SS nanocomposites using the experimental and simulation methods. They found that the volumetric energy densities directly affected the pores and defects due to the thermo-kinetics and thermo-capillary characteristics within the molten pool. As indicated by Yusuf et al. [40], the laser energy absorbed per unit area decreases with increasing hatch spacing. For a given layer, an extremely small or large track space implies different laser energy inputs, which may worsen the surface quality and increase the number of defects. By combining the results in Figure 10 and Figure 11, it is reasonable to conclude that increasing the hatch spacing can improve the building rate and decrease the relative density of the parts fabricated by SLM. Consequently, it is necessary to consider other factors, such as laser power, scan speed, hatch spacing, etc., to optimize the process parameters.

## 4. Conclusions

This study presented a combined simulation and experimental approach to investigate the influence of hatch spacing on the melt pool and as-built quality of 316L stainless steel samples fabricated by SLM. The general conclusions can be summarized as follows.

- Increasing the hatch spacing reduces the maximum temperature and heat accumulation. When the hatch spacing increased from 75 to 200 μm, the simulated width of the melt pool decreased from 221 to 212 μm, while the depth of the melt pool increased from 81.4 to 85.2 μm. The finite element analysis model also managed to predict the microstructure and melt pool sizes based on the cooling rate. The simulation results were in good agreement with the experimental data.
- The simulation models were used for several hatch spacings. The solidified development at the end of the fifth track was clearly demonstrated. The overlap rate between adjacent tracks was investigated by numerical simulations. A reasonable value of the hatch spacing of 100 μm was revealed, which yielded a relatively flat sintering condition and an appropriate overlap rate (approximately 54%) between adjacent tracks. Similarly, the experiments also confirmed the simulation results.
- The hatch spacing had a strong influence on the surface quality and relative density of the SLM-fabricated 316L SS samples. The experimental results showed that, with an appropriate hatch spacing of 100 μm, samples with a high relative density of 99.9% and low average surface roughness of 2.68 μm could be fabricated by SLM. These findings provide significant insight into the optimization of SLM process parameters.

## Figures and Tables

**Table 1:** Properties of the material (316L SS) and SLM processing conditions.

Parameter	Value
Laser power, P (W)	200
Layer thickness, d (μm)	50
Diameter of laser beam, D (μm)	75
Scan speed, V (mm/s)	400
Hatch spacing, H (μm)	75, 100, 150, 200
Absorption rate of powder, A	0.33
Latent heat of fusion (kJ/kg)	273

**Table 2:** Thermo-physical parameters of 316L SS [26,27].

**Temperature, T (K)**	293	633	1073	1353	1713	2073
**Thermal conductivity, k_s_ [W/(m∙K)]**	13.1	16.3	22.5	28.9	35.5	18.9
**Specific heat capacity, c [J/(kg∙K)]**	472	505	562	680	822	820
**Density,** $\rho_{s}$ **(kg/m^3^)**	7.9	7.8	7.7	7.5	7.4	7.1

**Table 3:** Chemical composition of 316L SS.

Element	Fe	Cr	Ni	Mo	Mn	Si	N	O	P	C	S
wt%	Balance	16–18	11–13	2.5	1.5	0.8	0.1	0.1	0.04	0.03	0.02

**Table 4:** Summary of grain size based on simulation and SEM micrographs.

Sample (Hatch Spacing, μm)	Ṫ (10^5^ K/s)	λ_FEM_ (μm)	λ_SEM_ (μm)
75	2.62	1.25	0.86
100	2.44	1.28	0.95
150	2.23	1.32	1.08
200	2.18	1.33	1.15
